# On the Vibrations and Stability of Moving Viscoelastic Axially Functionally Graded Nanobeams

**DOI:** 10.3390/ma13071707

**Published:** 2020-04-06

**Authors:** Ali Shariati, Dong won Jung, Hamid Mohammad-Sedighi, Krzysztof Kamil Żur, Mostafa Habibi, Maryam Safa

**Affiliations:** 1 Division of Computational Mathematics and Engineering, Institute for Computational Science, Ton Duc Thang University, Ho Chi Minh City 758307, Vietnam; alishariati@tdtu.edu.vn; 2 Faculty of Civil Engineering, Ton Duc Thang University, Ho Chi Minh City 758307, Vietnam; 3 School of Mechanical Engineering, Jeju National University, Jeju, Jeju-do 690-756, Korea; 4 Mechanical Engineering Department, Faculty of Engineering, Shahid Chamran University of Ahvaz, Ahvaz 61357-43337, Iran; h.msedighi@scu.ac.ir; 5 Faculty of Mechanical Engineering, Bialystok University of Technology, 15-351 Bialystok, Poland; 6 Center of Excellence in Design, Robotics and Automation, Department of Mechanical Engineering, Sharif University of Technology, Azadi Avenue, P.O. Box 11365-9567, Tehran, Iran; Habibi_mech@yahoo.com; 7 Institute of Research and Development, Duy Tan University, Da Nang 550000, Vietnam

**Keywords:** axially graded materials, viscoelastic materials, axially moving nanobeams, stability map, divergence and flutter analysis

## Abstract

In this article, size-dependent vibrations and the stability of moving viscoelastic axially functionally graded (AFG) nanobeams were investigated numerically and analytically, aiming at the stability enhancement of translating nanosystems. Additionally, a parametric investigation is presented to elucidate the influence of various key factors such as axial gradation of the material, viscosity coefficient, and nonlocal parameter on the stability boundaries of the system. Material characteristics of the system vary smoothly along the axial direction based on a power-law distribution function. Laplace transformation in conjunction with the Galerkin discretization scheme was implemented to obtain the natural frequencies, dynamical configuration, divergence, and flutter instability thresholds of the system. Furthermore, the critical velocity of the system was evaluated analytically. Stability maps of the system were examined, and it can be concluded that the nonlocal effect in the system can be significantly dampened by fine-tuning of axial material distribution. It was demonstrated that AFG materials can profoundly enhance the stability and dynamical response of axially moving nanosystems in comparison to homogeneous materials. The results indicate that for low and high values of the nonlocal parameter, the power index plays an opposite role in the dynamical behavior of the system. Meanwhile, it was shown that the qualitative stability of axially moving nanobeams depends on the effect of viscoelastic properties in the system, while axial grading of material has a significant role in determining the critical velocity and natural frequencies of the system.

## 1. Introduction

Axially moving systems have a broad spectrum of applications in various engineering fields, especially in nanoscience and nanotechnology such as subminiature belts, silicon acceleration sensors, and nanowires [1,2]. Accordingly, mathematical modeling and vibrational analysis of these applicable structures have attracted much attention in recent decades [3,4,5]. In this field, a limited number of experimental studies have been performed and compared with the theoretical analyses [6,7]. These investigations revealed that engineers could appropriately trust the results of computer simulations and mathematical modeling techniques [8,9,10]. For instance, Tan and Ying [11] theoretically and experimentally investigated the active control of the axially moving strings with various boundary conditions. They presented the closed-form expression for the lateral vibration of the system. Additionally, they validated the stability and effectiveness of the proposed controller through both experiments and computer simulations. Duan et al. [12] studied the dynamics of an axially moving nested cantilevered beam with a tip mass. Utilizing the modified Galerkin approach, they solved the partial differential equation of motion of the structure. They calculated the acceleration responses of the system during both extension and retraction. Additionally, they observed that the proposed theoretical model could be validated by experimental data. Hayes and Bhushan [13] developed a vibration model for a doubly clamped Euler–Bernoulli beam with axial velocity. They probed the effect of length, thickness, tension, and axial velocity on the vibration characteristics of the system.

In the determination of dynamical response and the stability of axially moving continua, system velocity is a crucial parameter, so at different velocities, various instability mechanisms such as divergence and flutter occur [14]. Preventing instability and eliminating excessive vibrations are necessary engineering requirements in axially moving nanosystems. To this end, numerous studies have been conducted to explore the influences of different parameters on the stability and vibrational behavior of these structures. For instance, Dehrouye-Semnani et al. [15] examined the stability and vibration of axially moving Euler–Bernoulli and Timoshenko microbeams with constant velocity. Their results illustrated that when the length scale parameter of the system is relatively equal to the thickness of the microbeam, the natural frequency, stability, and critical velocity of the system dramatically enhanced. Liu et al. [16] surveyed the dynamical response and instability thresholds of axially moving nanobeams with time-dependent velocity. They concluded that the natural frequency of the system first decreases slightly, and then increases rapidly with an increase in the system velocity. Wang et al. [17] utilized complex modal analysis to evaluate the role of modal truncation order on the transverse free vibration response of axially moving nanobeams by considering von Karman geometric nonlinearity. They declared that the natural frequencies of the nanobeam have a significant dependency on the size effect of the mechanical properties of the system. Wang et al. [18] analyzed the nonlinear dynamical behavior of axially moving nanobeam using Eringen’s nonlocal two-phase integral model. Their outcomes demonstrated that the vibrational frequency and critical velocity of the system ascend by increasing the geometric parameter for various boundary conditions. Stability and nonlinear vibration of axially moving viscoelastic Rayleigh nanobeams with time-dependent velocity based on higher-order nonlocal elasticity theory were carried out by Rezaee and Lotfan [19]. They revealed that the instability boundaries could be greatly affected by the nonlocal parameter, so the second bifurcation point occurs before the first one. Kiani [20] explored the shear and surface effects on the stability regions and dynamics of axially moving nanobeams and presented an explicit expression for the critical velocity of the system through an analytical approach. According to the nonlocal strain gradient theory, Gou et al. [21] investigated the Vibro-buckling and vibration characteristics of nanobeams by considering the axial motion or rotational motion. They showed that the critical velocity of the structure promotes by ascending the strain gradient parameter. In a majority of available reports in the literature, the axially moving nanostructure materials are considered to be homogeneous and uniform. Recently, engineers have promoted the performance of dynamical systems by improving the material properties obtained through technological progress [22,23,24]. Recently, a few studies have been conducted to find possible ways to enhance the performance of axially moving systems by designing nonuniformity in the physical and geometrical properties of the system [25].

Functionally graded (FG) materials are a class of composite materials that provide desirable characteristics for special and complicated applications by smooth and continuous gradation of mechanical characteristics along a specific direction [26]. Compared with isotropic and conventional laminated composites, FG materials are used in industrial areas due to their valuable advantages such as better corrosion resistance, higher fracture stiffness, and lower stress concentration [27]. Hence, employing FG materials in axially moving macro/nanosystems offers several advantages. For instance, Piovan and Sampaio [28] inspected the dynamical response of axially moving FG Euler–Bernoulli beams made of ceramic and metal by employing the finite element method. Their simulation results demonstrated that the vibrational frequencies of the structure were higher when the structure is mainly composed of a ceramic substrate. Sui et al. [29] addressed the influence of different grading functions on the transverse free vibration of axially moving FG Timoshenko beams. Additionally, they focused on the differences between the vibrational behavior of Timoshenko and Euler–Bernoulli beams through numerical examples. Utilizing the Galerkin method in conjunction with Eringen’s nonlocal theory, Kiani [30] analyzed the longitudinal and transverse free vibration of axially moving FG Rayleigh nanobeam and determined the flutter and divergence instability thresholds of the system for various parameters such as length of the nanobeam and nonlocal parameter. Recently, Yan et al. [31] accomplished the nonlinear vibration of axially moving FG beams by considering the influence of geometric nonlinearity and axial force. They reported the conditions of occurrence of subharmonic resonance in the system by applying the direct multiscale method.

To the best of the authors’ knowledge, in all of the investigations that focused on the dynamics of axially moving FG nanobeams, it is supposed that the system materials are graded in the thickness direction. While despite the importance of the gradation of material properties along the axial direction, the vibrations and stability of axially moving AFG nanobeams have not been reported in the literature yet. Hence, in this article, the dynamic analysis and stability improvement of axially moving AFG pinned–pinned nanobeams are investigated. It was assumed that the material characteristics of the system varied along the axial direction according to a power-law function. The dynamical equation of the system was obtained by utilizing Hamilton’s principle. The reduced-order equation of motion was acquired by the Laplace transformation in conjunction with the Galerkin technique. The natural frequencies and stability thresholds of the system were determined numerically. Additionally, an analytical expression is presented for the critical velocity of the structure. Finally, the influence of the gradation of material properties, nonlocal parameter, and system velocity on the dynamical response and stability regions of the system are illuminated.

## 2. Problem Formulation

**Figure 1:**
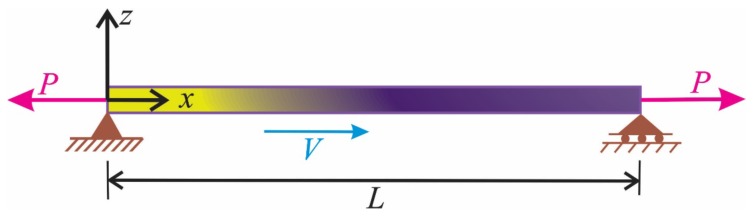
Schematic view of a moving axially functionally graded (AFG) nanobeam.

Figure 1 shows the schematic of an axially moving AFG nanobeam. The supported nanobeam is moving with constant velocity (*v*) in the axial direction and subjected to an axial tension load (*P*). Length, surface area, and inertia moment of the nanobeam are indicated by *L*, *A*, and *I*, respectively.

It is assumed that density (*ρ*(*x*)) and elastic modulus (*E*(*x*)) of the system are graded in the longitudinal direction as follows

(1) $$\rho \left(x\right)=\rho_{0}g\left(x\right)$$

(2) $$E\left(x\right)=E_{0}f\left(x\right)$$

where

(3) $$g\left(x\right)=1+\left(\alpha_{\rho }-1\right){\left(\frac{x}{L}\right)}^{k}$$

(4) $$f\left(x\right)=1+\left(\alpha_{E}-1\right){\left(\frac{x}{L}\right)}^{k}$$

where *k* is the power index. Additionally, *α*_E_ and *α_ρ_* represent the elastic modulus and density gradient parameters, respectively, which are defined as the ratio of the material properties at the end of the nanobeam (*x* = *L*) to those of the first of the nanobeam (*x* = 0):

(5) $$\alpha_{\rho }=\frac{\rho_{L}}{\rho_{0}}$$

(6) $$\alpha_{E}=\frac{E_{L}}{E_{0}}$$

As reported in the literature [32,33,34], the obtained results of the modeling of ultra-small scale structures based on classical theories do not have acceptable accuracy. Additionally, experimental studies have proven that classical continuum theories are incapable of predicting the mechanical behavior of the small-scale structures [35]. As a result, to capture the size effects of the material of nanosized structures, employing higher-order elasticity theories is essential in the mathematical modeling of these structures [36,37]. The nonlocal elasticity theory is one of the applicable non-classical theories that incorporate nanostructure-dependent size effects [38]. The impact of the nonlocal parameter on the dynamical characteristics of nano-mechanical systems has been extensively elaborated by numerous researchers [39]. To capture the small-scale effects, the nonlocal elasticity beam model is considered. To this end, by considering the constitutive equation of the viscoelastic material according to the Kelvin–Voight model and the linear strain–displacement relation, one can express [19,40,41,42]:

(7) $$\varepsilon_{x}=-z\frac{\partial^{2}w}{\partial x^{2}}$$

(8) $$\sigma_{x}^{\mathrm{nl}}-{\left(e_{0}a\right)}^{2}\frac{\partial^{2}\sigma_{x}^{\mathrm{nl}}}{\partial x^{2}}=E\left(x\right)\varepsilon_{x}+\beta \frac{D}{Dt}\varepsilon_{x}$$

where *β*, *ε*_x_, $\sigma_{x}^{\mathrm{nl}}$, and *w*(*x*, *t*) represent the viscosity coefficient, axial strain, nonlocal axial stress, and transverse displacement of the system, respectively. Additionally, *e*_0_ is the material constant. Furthermore, *a* is the characteristic length. The resultant nonlocal bending moment can be defined as [43,44]:

(9) $$M^{\mathrm{nl}}=-\int_{A}^{}z\sigma_{x}^{\mathrm{nl}}dA$$

Substituting Equations (7) and (8) into Equation (9) yields the following relation:

(10) $$M^{\mathrm{nl}}-{\left(e_{0}a\right)}^{2}\frac{\partial^{2}M^{\mathrm{nl}}}{\partial x^{2}}=E\left(x\right)I\frac{\partial^{2}w}{\partial x^{2}}+\beta I\left(\frac{\partial^{3}w}{\partial x^{2}\partial t}+V\frac{\partial^{3}w}{\partial x^{3}}\right)$$

The kinetic and potential energies of the system can be expressed as follows [14,19,45]:

(11) $$T=\frac{1}{2}\int_{0}^{L}\rho \left(x\right)A\left(V^{2}+{\left(\dot{w}+Vw^{\prime }\right)}^{2}\right)dx$$

(12) $$U=\frac{1}{2}\int_{0}^{L}\left(P{\left(w^{\prime }\right)}^{2}+M^{\mathrm{nl}}w^{\prime \prime }\right)dx$$

where primes and dots represent the spatial and temporal derivatives. The governing dynamical equation of motion can be obtained by employing Hamilton’s principle as follows [46,47]:

(13) $$\begin{array}{l} p(x)A\left(\ddot{w}+2V{\dot{w}}^{\prime }+V^{2}w^{\prime \prime }\right)+\rho^{\prime }\left(x\right)A\left(V\dot{w}+V^{2}w^{\prime }\right)-Pw^{\prime \prime }\\ \;-{\left(e_{o}a\right)}^{2}(\rho^{\prime \prime }\left(x\right)A\left(\ddot{w}+2V{\dot{w}}^{\prime }+V^{2}w^{\prime \prime }\right)\\ \;+\rho^{\prime }\left(x\right)A\left({\ddot{w}}^{\prime }+2V{\dot{w}}^{\prime \prime }+V^{2}w^{\prime \prime \prime }\right)\\ \;+2\rho^{\prime }\left(x\right)A\left({\ddot{w}}^{\prime }+2V{\dot{w}}^{\prime \prime }+V^{2}w^{\prime \prime \prime }\right)+\rho^{\prime \prime \prime }\left(x\right)A\left(V\dot{w}+V^{2}w^{\prime }\right)\\ \;+2\rho^{\prime \prime }\left(x\right)A\left(V{\dot{w}}^{\prime }+V^{2}w^{\prime \prime }\right)+\rho^{\prime }\left(x\right)A\left(V{\dot{w}}^{\prime \prime }+V^{2}w^{\prime \prime \prime }\right)-Pw^{⁗})\\ \;+E\left(x\right)Iw^{⁗}+2E^{\prime }\left(x\right)Iw^{\prime \prime \prime }+E^{\prime \prime }\left(x\right)Iw^{\prime \prime }+\beta I\left({\dot{w}}^{⁗}+Vw^{⁗}{}^{\prime }\right)\\ \;=0 \end{array}$$

To derive the non-dimensional dynamical equation of motion, the following normalized parameters are introduced to derive dimensionless equations [48]:

(14) $$\xi =\frac{x}{L},\;\;\eta =\frac{w}{L},\;\;\tau =\frac{t}{T}$$

where

(15) $$T=\sqrt{\frac{\rho_{0}AL^{2}}{P}}$$

Substituting dimensionless parameters of Equation (14) into Equation (13), one can acquire the dimensionless dynamical equation to become:

(16) $$\begin{array}{l} g\left(\xi \right)\left(\ddot{\eta }+2v{\dot{\eta }}^{\prime }+v^{2}\eta^{\prime \prime }\right)+g^{\prime }\left(\xi \right)\left(v\dot{\eta }+v^{2}\eta^{\prime }\right)-\eta^{\prime \prime }\\ \;-\mu^{2}(g^{\prime \prime }\left(\xi \right)\left(\ddot{\eta }+2v{\dot{\eta }}^{\prime }+v^{2}\eta^{\prime \prime }\right)+2g^{\prime }\left(\xi \right)\left({\ddot{\eta }}^{\prime }+2v{\dot{\eta }}^{\prime \prime }+v^{2}\eta^{\prime \prime \prime }\right)\\ \;+g\left(\xi \right)\left({\ddot{\eta }}^{\prime \prime }+2v{\dot{\eta }}^{\prime \prime \prime }+v^{2}\eta^{⁗}\right)+g^{\prime \prime \prime }\left(\xi \right)\left(v\dot{\eta }+v^{2}\eta^{\prime }\right)\\ \;+2g^{\prime \prime }\left(\xi \right)\left(v{\dot{\eta }}^{\prime }+v^{2}\eta^{\prime \prime }\right)+g^{\prime }\left(\xi \right)\left(v{\dot{\eta }}^{\prime \prime }+v^{2}\eta^{\prime \prime \prime }\right)-\eta^{⁗})\\ \;+k_{f}^{2}\left(f\left(\xi \right)\eta^{⁗}+2f^{\prime }\left(\xi \right)\eta^{\prime \prime \prime }+f^{\prime \prime }\left(\xi \right)\eta^{\prime \prime }\right)+\zeta \left({\dot{\eta }}^{⁗}+v\eta^{⁗}{}^{\prime }\right)=0 \end{array}$$

The dimensionless parameters appeared in Equation (16) are defined as

(17) $$v=V\sqrt{\frac{\rho_{0}A}{P}},\;\;k_{f}=\sqrt{\frac{E_{0}I}{PL^{2}}},\;\;\mu =\frac{e_{o}a}{L},\;\;\zeta =\frac{\beta I}{PL^{3}}\sqrt{\frac{P}{\rho_{0}A}}$$

where *k*_f_, *v*, *μ*, and *ζ* represent the dimensionless flexural stiffness, dimensionless axial velocity, nonlocal parameter, and viscosity coefficient, respectively.

## 3. Numerical Procedure

According to the Laplace transform, one can write [49]:

(18) $$L\left[\eta^{\left(\varepsilon \right)}\left(\tau \right)\right]=s^{\varepsilon }\eta \left(s\right)-s^{\varepsilon -1}\eta \left(0\right)$$

Hence, the dimensionless governing equation of motion in the Laplace domain can be rewritten as:

(19) $$\begin{array}{l} g\left(\xi \right)\left(s^{2}\eta +2sv\eta^{\prime }+v^{2}\eta^{\prime \prime }\right)+g^{\prime }\left(\xi \right)\left(sv\eta +v^{2}\eta^{\prime }\right)-\eta^{\prime \prime }\\ \;-\mu^{2}(g^{\prime \prime }\left(\xi \right)\left(s^{2}\eta +2sv\eta^{\prime }+v^{2}\eta^{\prime \prime }\right)\\ \;+2g^{\prime }\left(\xi \right)\left(s^{2}\eta^{\prime }+2sv\eta^{\prime \prime }+v^{2}\eta^{\prime \prime \prime }\right)\\ \;+g\left(\xi \right)\left(s^{2}\eta^{\prime \prime }+2sv\eta^{\prime \prime \prime }+v^{2}\eta^{⁗}\right)+g^{\prime \prime \prime }\left(\xi \right)\left(sv\eta +v^{2}\eta^{\prime }\right)\\ \;+2g^{\prime \prime }\left(\xi \right)\left(sv\eta^{\prime }+v^{2}\eta^{\prime \prime }\right)+g^{\prime }\left(\xi \right)\left(sv\eta^{\prime \prime }+v^{2}\eta^{\prime \prime \prime }\right)-\eta^{⁗})\\ \;+k_{f}^{2}\left(f\left(\xi \right)\eta^{⁗}+2f^{\prime }\left(\xi \right)\eta^{\prime \prime \prime }+f^{\prime \prime }\left(\xi \right)\eta^{\prime \prime }\right)+\zeta \left({\dot{\eta }}^{⁗}+v\eta^{⁗}{}^{\prime }\right)=0 \end{array}$$

To discretize Equation (19), the transverse displacement of the nanobeam can be assumed as follows [50,51,52]:

(20) $$\eta \left(\xi ,s\right)=\overset{n}{\underset{r=1}{\sum }}\varphi_{r}\left(\xi \right)q_{r}\left(s\right)$$

where *n*, *φ_r_*, and *q*_r_ indicate the number of basic functions, dimensionless mode shapes of the pinned–pinned nanobeam, and dimensionless generalized coordinate, respectively. The normalized mode shapes of a simply supported nanobeam are given by [53]

(21) $$\varphi_{r}\left(\xi \right)=\sqrt{2}\sin\left(\sigma_{r}\xi \right)$$

where

(22) $$\sigma_{r}=p_{j}\sqrt{\frac{\sqrt{4+{\left(\mu p_{j}\right)}^{4}}+{\left(\mu p_{j}\right)}^{2}}{2}}$$

The characteristic frequency equation of the simply supported boundary condition is as follows [54]:

(23) $$\sin\left(\sigma_{r}\right)=0$$

Inserting Equations (20)–(22) into Equation (19), multiplying by *φ_s_*, integrating over the nanobeam length and considering orthogonality condition of mode shapes leads to the following relation:

(24) $$Z_{\mathrm{mn}}=K_{\mathrm{mn}}+C_{\mathrm{mn}}+M_{\mathrm{mn}}$$

where Z denotes the coefficient matrix and M, C, and K are defined as follows:

(25) $$M_{jk}=-s^{2}\mu^{2}\left(\int_{0}^{1}g^{\prime \prime }\left(\xi \right)\phi_{j}\left(\xi \right)\phi_{k}\left(\xi \right)d\xi +2\int_{0}^{1}g^{\prime }\left(\xi \right)\phi_{j}\left(\xi \right)\phi_{k}^{’}\left(\xi \right)d\xi +\int_{0}^{1}g\left(\xi \right)\phi_{j}\left(\xi \right)\phi_{k}^{’’}\left(\xi \right)d\xi \right)+s^{2}\int_{0}^{1}g\left(\xi \right)\phi_{j}\left(\xi \right)\phi_{k}\left(\xi \right)d\xi$$

(26) $$\begin{array}{l} C_{jk}=sv(2\int_{0}^{1}g\left(\xi \right)\phi_{j}\left(\xi \right)\phi_{k}^{\prime }\left(\xi \right)d\xi +\int_{0}^{1}g^{\prime }\left(\xi \right)\phi_{j}\left(\xi \right)\phi_{k}\left(\xi \right)d\xi \\ \;-\mu^{2}(2\int_{0}^{1}g^{\prime \prime }\left(\xi \right)\phi_{j}\left(\xi \right)\phi_{k}^{\prime }\left(\xi \right)d\xi +4\int_{0}^{1}g^{\prime }\left(\xi \right)\phi_{j}\left(\xi \right)\phi_{k}^{\prime \prime }\left(\xi \right)d\xi \\ \;+\int_{0}^{1}g^{\prime \prime \prime }\left(\xi \right)\phi_{j}\left(\xi \right)\phi_{k}\left(\xi \right)d\xi +2\int_{0}^{1}g^{\prime \prime }\left(\xi \right)\phi_{j}\left(\xi \right)\phi_{k}^{\prime }\left(\xi \right)d\xi \\ \;+\int_{0}^{1}g^{\prime }\left(\xi \right)\phi_{j}\left(\xi \right)\phi_{k}^{\prime \prime }\left(\xi \right)d\xi +2\int_{0}^{1}g\left(\xi \right)\phi_{j}\left(\xi \right)\phi_{k}^{\prime \prime \prime }\left(\xi \right)d\xi ))\\ \;+s\zeta \int_{0}^{1}\phi_{s}\left(\xi \right)\phi_{r}^{⁗}\left(\xi \right)dx \end{array}$$

(27) $$\begin{array}{l} K_{jk}=v^{2}(\int_{0}^{1}g\left(\xi \right)\phi_{j}\left(\xi \right)\phi_{k}^{\prime \prime }\left(\xi \right)+\int_{0}^{1}g^{\prime }\left(\xi \right)\phi_{j}\left(\xi \right)\phi_{k}^{\prime }\left(\xi \right)d\xi \\ \;-\mu^{2}(\int_{0}^{1}g^{\prime \prime }\left(\xi \right)\phi_{j}\left(\xi \right)\phi_{k}^{\prime \prime }\left(\xi \right)d\xi +2\int_{0}^{1}g^{\prime }\left(\xi \right)\phi_{j}\left(\xi \right)\phi_{k}^{\prime \prime \prime }\left(\xi \right)d\xi \\ \;+\int_{0}^{1}g\left(\xi \right)\phi_{j}\left(\xi \right)\phi_{k}^{⁗}\left(\xi \right)d\xi +\int_{0}^{1}g^{\prime \prime \prime }\left(\xi \right)\phi_{j}\left(\xi \right)\phi_{k}^{\prime }\left(\xi \right)d\xi \\ \;+2\int_{0}^{1}g^{\prime \prime }\left(\xi \right)\phi_{j}\left(\xi \right)\phi_{k}^{\prime \prime }\left(\xi \right)d\xi +\int_{0}^{1}g^{\prime }\left(\xi \right)\phi_{j}\left(\xi \right)\phi_{k}^{\prime \prime \prime }\left(\xi \right)d\xi ))\\ \;+k_{f}^{2}\int_{0}^{1}\left(f\left(\xi \right)\phi_{j}\left(\xi \right)\phi_{k}^{⁗}\left(\xi \right)+2f^{\prime }\left(\xi \right)\phi_{j}\left(\xi \right)\phi_{k}^{\prime \prime \prime }\left(\xi \right)+f^{\prime \prime }\left(\xi \right)\phi_{j}\left(\xi \right)\phi_{k}^{\prime \prime }\left(\xi \right)\right)d\xi \\ \;-\int_{0}^{1}\phi_{j}\left(\xi \right)\phi_{k}^{\prime \prime }\left(\xi \right)d\xi +\mu^{2}\int_{0}^{1}\phi_{j}\left(\xi \right)\phi_{k}^{⁗}\left(\xi \right)d\xi +\zeta v\int_{0}^{1}\phi_{s}\left(\xi \right)\phi_{r}^{\prime \prime \prime }{}^{\prime \prime }\left(\xi \right)d\xi \end{array}$$

## 4. Stability Analysis

For the existence of non-trivial solutions, the determinant of the coefficient matrix must be equal to zero

(28) det[Z(s)]=0

The complex-valued roots (*λ*) of Equation (28) are the natural frequency and can be computed in terms of system velocity, nonlocal parameter, viscosity coefficient, elastic modulus, and density gradient parameters. It should be mentioned that the system experiences divergence instability when the imaginary part of one of the natural frequencies becomes zero (*ω* = Image(*λ*) = 0). Additionally, flutter instability occurs in the system when the real part of one of the natural frequencies becomes negative (*δ* = Real(*λ*) < 0) [55].

## 5. Results and Discussion

In this section, first, the eigenvalues and mode shapes for a simply-supported nanobeam are calculated and compared with the available theoretical results. Then, the results for the homogeneous system are extracted and compared with those available in the literature. Afterward, the effect of axial load, nonlocal parameter, viscosity, power index, elastic modulus, and density gradient parameters on the natural frequencies, dynamical response, and stability boundaries of the simply supported system are evaluated. It should be mentioned that the dynamical response of the system can be determined by applying the fourth-order Runge–Kutta technique.

### 5.1. Model Validation

**Figure 2:**
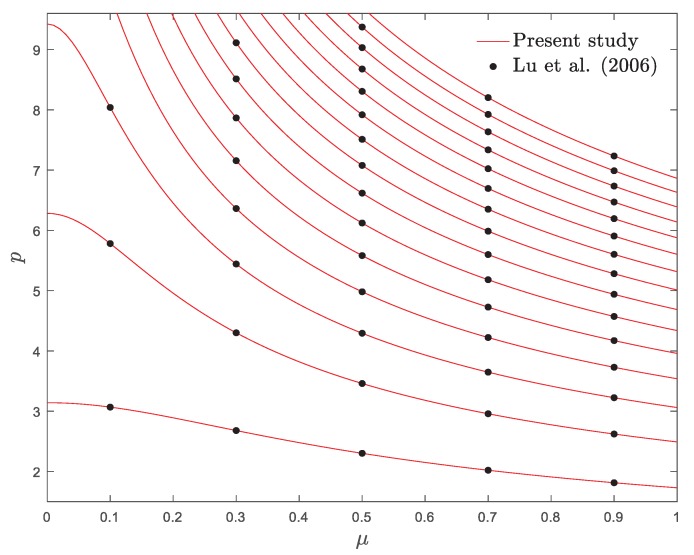
Variations of fifteen eigenvalues of a pinned–pinned nanobeam against nonlocal parameter.

**Figure 3:**
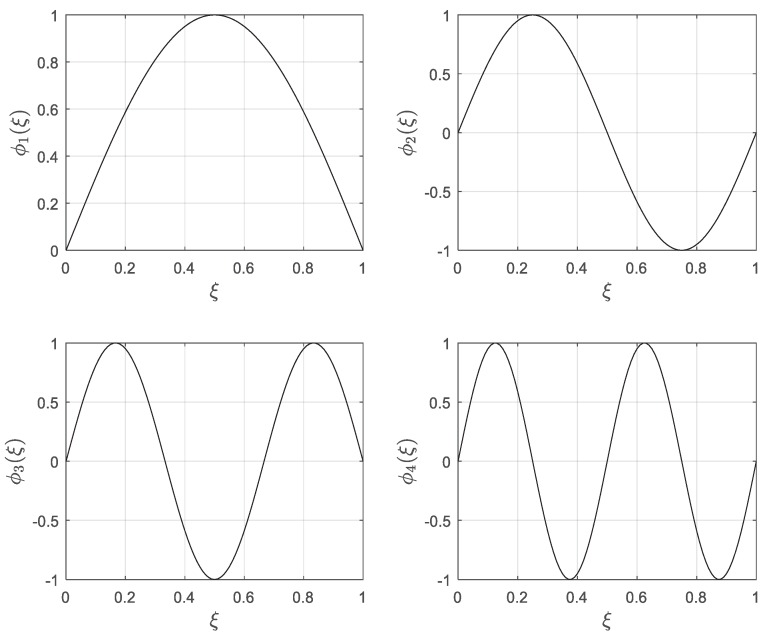
First four mode shapes of a pinned–pinned nanobeam for *μ* = 0.4.

The first fifteen eigenvalues as well as first four mode shapes of a pinned–pinned nanobeam are depicted in Figure 2 and Figure 3, respectively. As shown, the numerical results demonstrated an acceptable correlation with those in [54]. According to the nonlocal model, the eigenvalues of the system descend with the increase of the nonlocal parameter.

**Figure 4:**
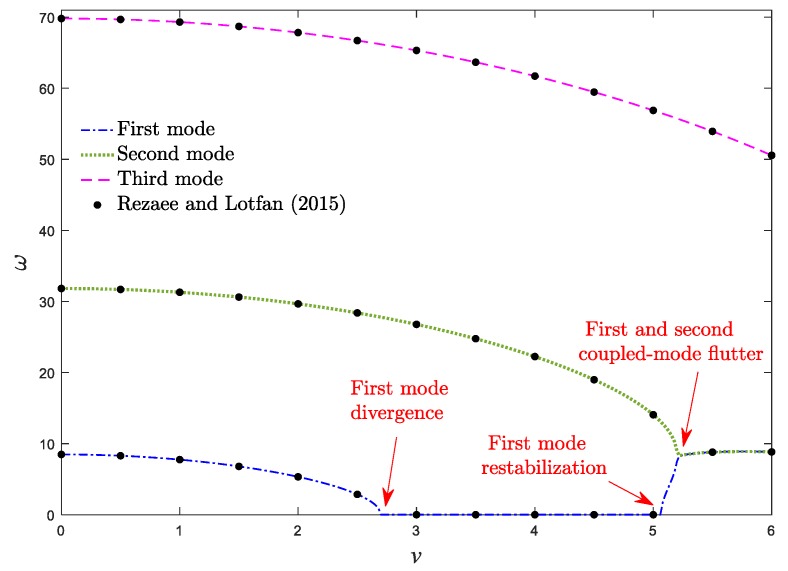
Real parts of the first three dimensionless vibrational frequencies of an axially moving isotropic nanobeam against the dimensionless velocity for *μ* = 0.025, *k*_f_ = 0.8, *ζ* = 0.

Figure 4 illustrates the first three natural frequencies of the axially moving homogeneous simply supported nanobeam with respect to the dimensionless axial velocity. It can be seen that the current results are in agreement with those reported by Rezaee and Lotfan [19].

### 5.2. Effect of Axial Load

**Figure 5:**
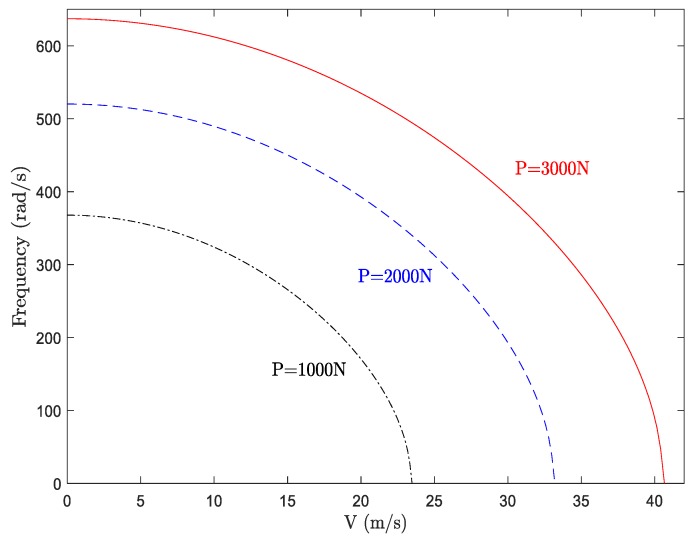
Fundamental frequency of an axially moving isotropic beam against axial velocity.

To illustrate the effect of axial load on the dynamics of the system, the fundamental frequency of the system against axial velocity is depicted in Figure 5 for different values of axial tension loads. The physical and geometrical parameters were considered as *E* = 200 Gpa, *ρ* = 7840 Kg/m^3^, *I* = 2 cm^4^, *L* = 0.2 m. As is evident, the increment of the axial tension load led to the enhancement of the effective stiffness of the system, and accordingly, the fundamental frequency and critical divergence of the system increased. Hence, one can state that at higher axial compressive loads, the moving system experiences the divergence (buckling) phenomenon at lower axial velocities.

### 5.3. Effect of Elastic Modulus Variation

**Figure 6:**
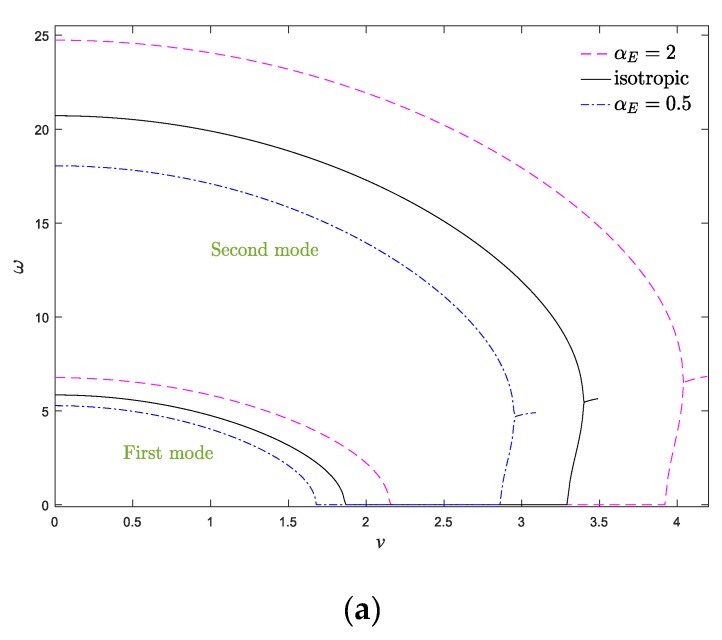

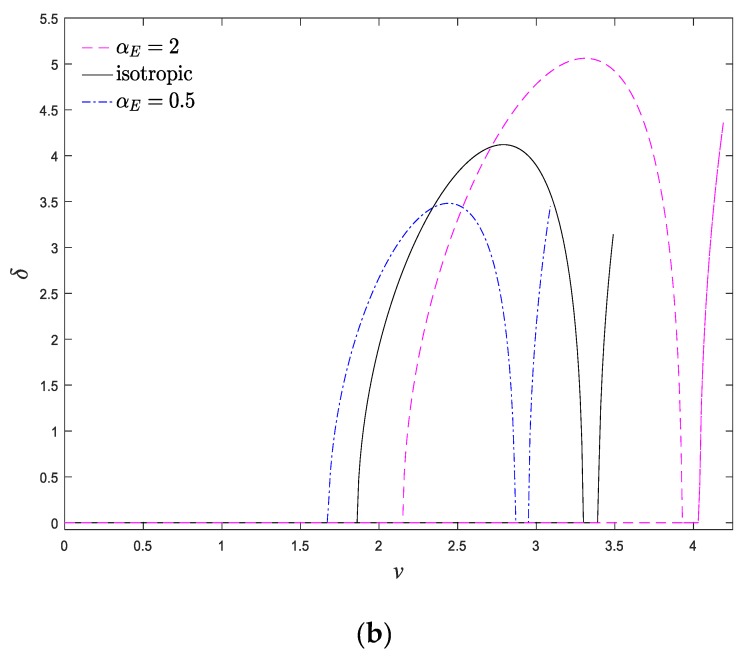
(**a**) Real and (**b**) imaginary parts of two first dimensionless natural frequency of the system against the dimensionless axial velocity for *ζ* = 0, *α_ρ_* = 1, *k*_f_ = 0.5, *μ* = 0, *k* = 1.

In Figure 6a,b, real and imaginary parts of the first two natural frequencies of the system versus axial velocity are shown, respectively, when *μ* = 0.025 and *k*_f_ = 0.5. As is obvious, the vibrational frequencies of the nanobeam were purely real when the axial velocity of the system was zero. Afterward, by increasing the velocity, the real part of the natural frequencies of the system declined gradually, while their imaginary part was still equal to zero. At the critical axial velocity (*u*_d_), the real part of the system frequencies vanished, and the system lost its stability and consequently underwent the divergence phenomenon. The induced divergence instability in moving structures due to ascending the axial velocity is analogous to that of buckling in classical beams under the compression load [14]. By further increasing the axial velocity, the fundamental frequency of the system became purely imaginary, while, the second natural frequency declined monotonically. Due to gyroscopic effects in the system, at higher velocities, the beam regained its stability again. In other words, the initiation and termination of divergence instability are related to the vanishing of real and imaginary parts of the fundamental frequency, respectively. Eventually, real parts of the first and second frequencies merge via a Paidoussis coupled-mode flutter bifurcation, while their imaginary parts increase and the system experiences flutter instability. In fact, in addition to velocities lower than critical velocity, a narrow velocity range (between the termination point of the divergence instability and the initiation of flutter instability) exists in which the system is stable at this operational velocity range. It should be mentioned that the system is no longer stable beyond the critical flutter velocity. As a result, the moving beam experiences a stability evolution of “stable—first mode divergence—stable—coupled-mode flutter”. According to Figure 6a, the real part of the system frequencies ascends by increasing the elastic modulus gradient parameter, particularly the frequencies of the higher modes. Additionally, increasing *α*_E_ leads to ascending the critical divergence and flutter velocities of the axially moving AFG nanobeam. In other words, it is feasible to hinder the occurrence of undesirable divergence phenomenon by increasing the elastic modulus gradient in moving nanostructures. This trend can be reasonable by this point, since the elastic modulus gradation parameter has an increasing role in the stiffness matrix; hence, any increment in the elastic modulus gradient leads to a stiffer structure and also wider stability regions. In other words, increasing *α*_E_ induces the stiffness-hardening effect in the system. Another important feature in Figure 6a,b is that the velocity bandwidth corresponding to the divergence and flutter phenomena in the system (*v*_d_ < *v* < *v*_f_) would be expanded by increasing *α*_E_. Moreover, based on these figures, compared with the exponential variation, the linear variation of the elastic modulus gradient leads to a more stable structure. As demonstrated in Figure 6b, the damping ratio of the system was higher for *α*_E_ > 1 and linear variation of elastic modulus. Accordingly, it is possible to determine the instability thresholds and vibrational behavior of the system by fine-tuning the elastic modulus gradient parameter. Generally, natural frequencies of the axially moving systems can be improved by ascending the elastic modulus in the longitudinal direction of the system in comparison to a homogeneous system.

**Figure 7:**
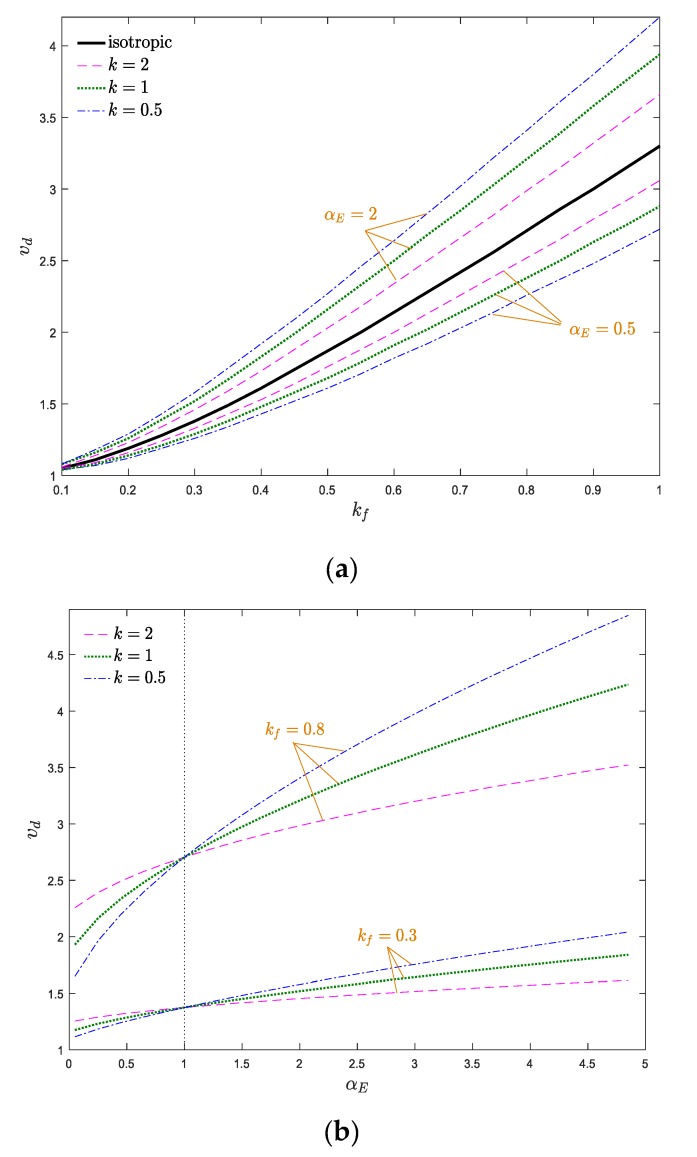
Dimensionless critical velocity of the system against (**a**) dimensionless flexural stiffness and (**b**) elastic modulus gradient parameter for *ζ* = 0, *α_ρ_* = 1, *μ* = 0.

To better understand the stable configuration of the system, stability maps of the system in *v*_d_–*k*_f_ and *v*_d_–*α*_E_ planes are drawn in Figure 7a,b, respectively. The indicated curves in the stability maps separate the stable and unstable zones, in which, above each curve, the structure is in the divergence instability condition. According to Figure 7a, the greater the flexural stiffness, the more stable the system becomes. Therefore, one can conclude that increasing the flexural stiffness has a stabilizing effect on the axially moving nanosystems. Additionally, by increasing the elastic modulus gradient parameter, the stability regions of the system expanded. According to Figure 7b, compared with the conventional homogeneous axially moving nanobeam (*α*_E_ = 1), the AFG nanosystem is more stable when *α*_E_ > 1 and ascending *α*_E_ promotes the stability of the system. It is evident from Figure 7a,b that the critical divergence velocity of the system increases by *k*_f_, which can be attributed to the stabilizing effects of the flexural stiffness. When *α*_E_ > 1, the increment/decrement of *k* leads to decrement/increment of the materials’ volume fraction at the end part of the nanobeam, which induces decrement/increment of system stiffness. Additionally, the effect of the elastic modulus gradient and power index variation are more prominent at higher values of *k*_f_ and the stability borders separate from each other. In other words, the variation of power index and elastic modulus gradient parameter play more important roles in the instability thresholds of the system at higher values of *k*_f_, and the stability borders of the system separate more from each other as *k*_f_ and *k* vary. Additionally, by approaching the value of the elastic modulus gradient parameter to one (homogeneous condition), the stability boundaries of the system are close to each other, and these boundaries separate from each other by increasing or decreasing of *α*_E_.

**Figure 8:**
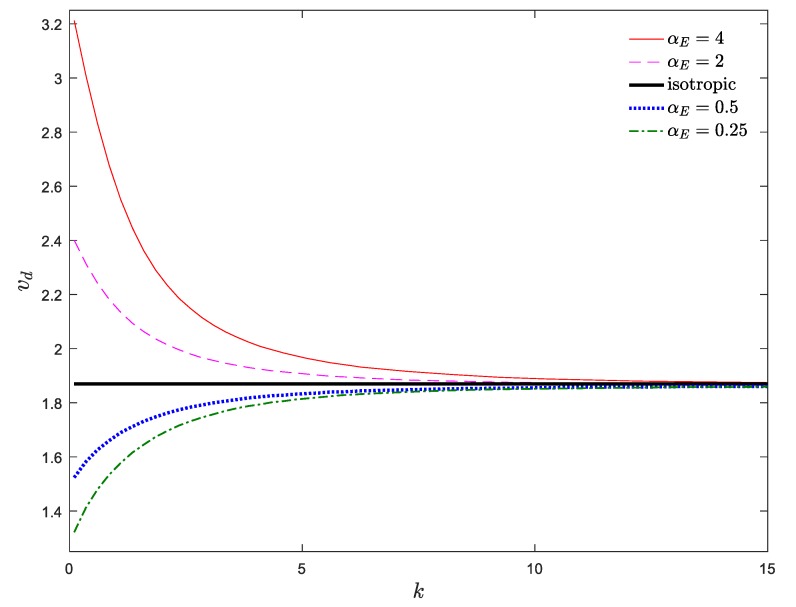
Dimensionless critical velocity of the system against power index for *ζ* = 0, *α_ρ_* = 1, *μ* = 0, *k*_f_ = 0.5.

The stability map of the system in the *v*_d_–*k* plane is demonstrated in Figure 8. The stability boundaries of the system in Figure 7 and Figure 8 are consistent with each other. As shown, when *α*_E_ > 1, a descending trend is observed by increasing *k*, while, this trend reverses for *α*_E_ < 1. In other words, descending the power index leads to a stiffer (softer) system for *α*_E_ > 1 (*α*_E_ < 1); hence, a decrement of the power index stabilizes (destabilizes) the system for *α*_E_ > 1 (*α*_E_ < 1). Moreover, a rapid change in the critical divergence velocity of an axially moving AFG nanobeam was observed for lower values of *k*, while for higher values of *k* (e.g., *k* > 10), the divergence velocity was practically constant, regardless of the value of *α*_E_ and converges to that of an axially moving homogenous nanobeam. As a result, it can be stated that the simultaneous selection of higher values of *α*_E_ and *k*_f_ as well as lower values of *k* is more appropriate for the stability enhancement of the system.

### 5.4. Effect of Density Gradient

**Figure 9:**
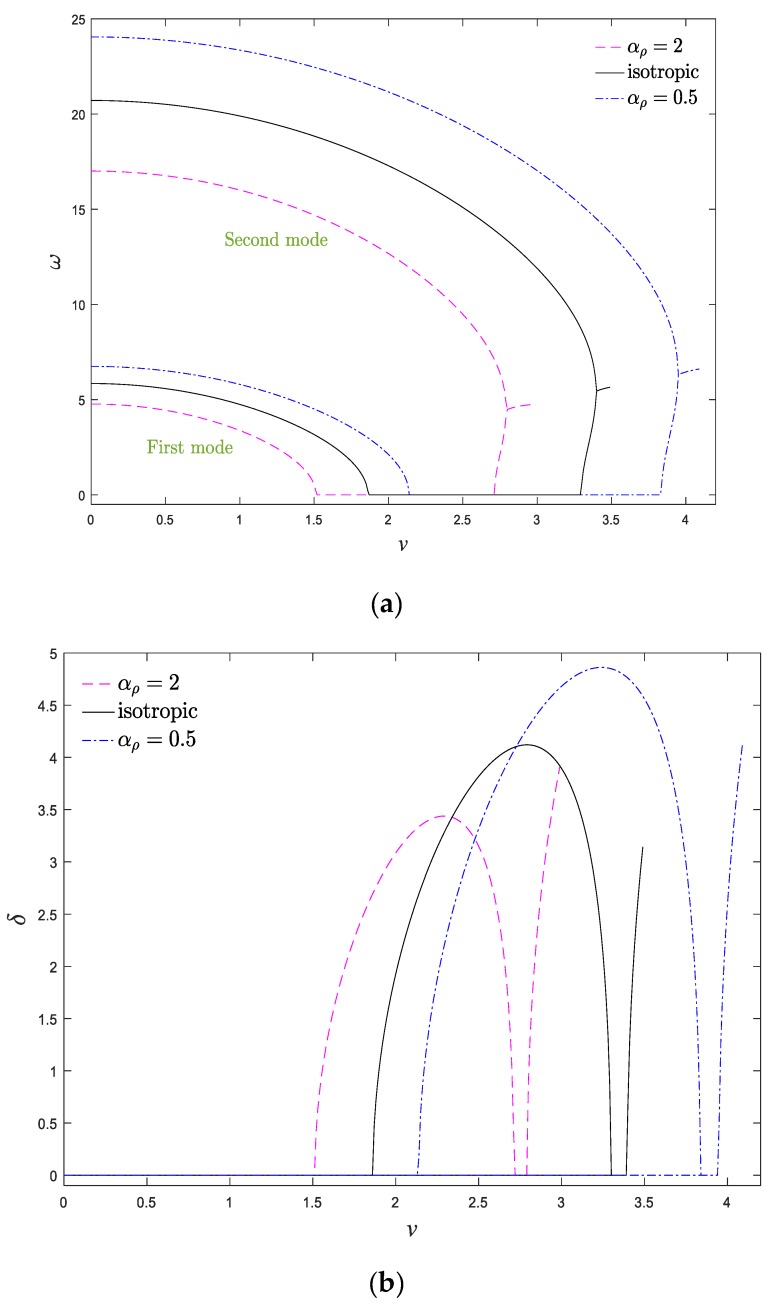
(**a**) Real and (**b**) imaginary parts of two first natural frequency of the system against the dimensionless axial velocity for *ζ* = 0, *α*_E_ = 1, *k*_f_ = 0.5, *μ* = 0, *k* = 1.

To explore the effect of the density gradient parameter on the system dynamics, the evolution of real and imaginary parts of first and second vibrational frequencies versus the axial velocity is demonstrated in Figure 9a,b for different density gradient parameters. The influence of the density gradient was more tangible in the natural frequencies of higher modes. It is evident that the effect of density and elastic modulus variations on the vibrational behavior are opposite to each other, so the natural frequencies of the system have a descending trend by increasing the density gradient parameter. Furthermore, the stability region shrinks by ascending *αρ*. The density gradient parameter contributed to the stiffness, damping, and mass matrices, which were associated with the mass-addition effect, gyroscopic effect, and stiffness-hardening effect, respectively. According to Figure 9, one can conclude that the mass-addition effect is dominant in the system. Another important feature in the frequency diagrams of moving AFG nanobeams is that compared with the case of density grading, the system experienced a wider range of frequencies in the case of elastic modulus grading. For this reason, from the perspective of design, the case of elastic modulus variation is more effective in avoiding the resonance phenomenon. By scrutinizing Figure 6 and Figure 9, it can be observed that axially grading the materials changes the critical velocities of the system, while it does not alter the order and the type of the system bifurcation series. Accordingly, one can conclude that the quantitative values of natural frequencies and critical velocities are strictly dependent on the axial grading of materials, but the qualitative stability of the system does not vary by the axial gradation of materials. According to Figure 6 and Figure 9, it can be mentioned that the vibrational behavior of the axially moving systems is highly dependent on the density, elastic modulus gradient parameters, and the type of their distributions. Furthermore, compared with the homogeneous systems, the AFG axially moving systems have higher natural frequencies when the density of the system descends along the axial direction.

**Figure 10:**
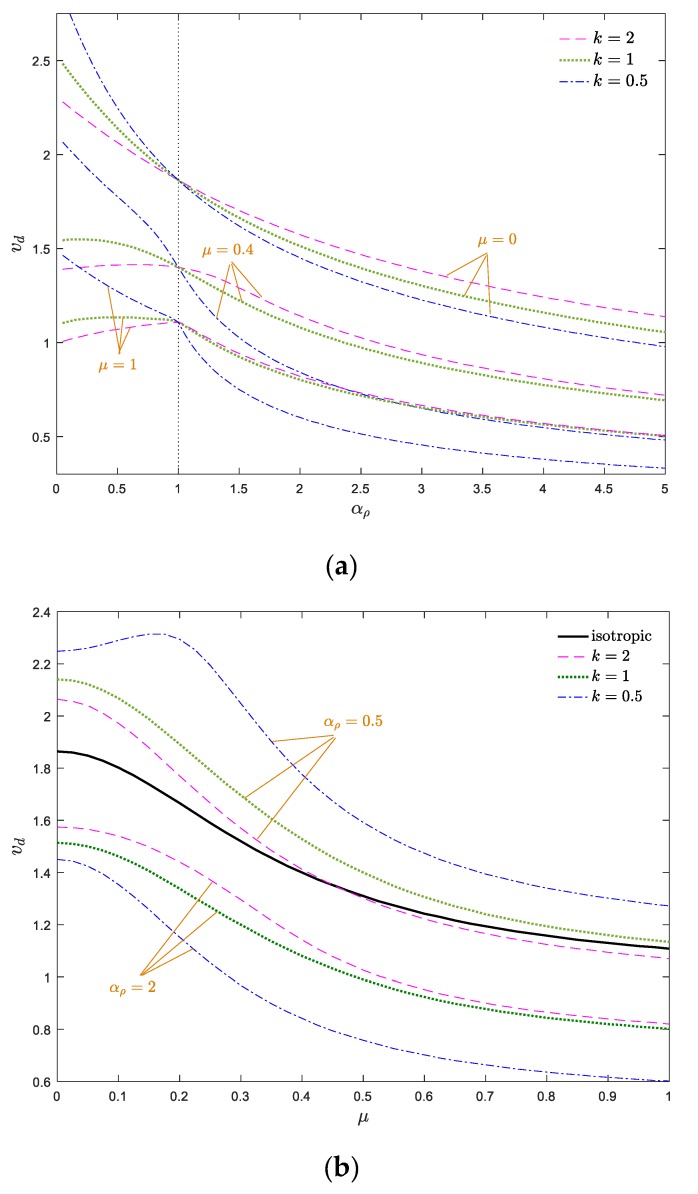
Dimensionless divergence velocity of a nanobeam against (**a**) density gradient parameter and (**b**) nonlocal parameter for *ζ* = 0, *α*_E_ = 1, *k*_f_ = 0.5.

To better describe the dynamical behavior of moving AFG nanobeams, the stability maps in *v*_d_–*α_ρ_* and *v*_d_–*μ* planes are plotted in Figure 10a,b, respectively. As demonstrated in Figure 10a, the moving AFG nanobeam is more stable for *α_ρ_* < 1 in comparison with the homogeneous one, especially at lower values of the nonlocal parameter. Additionally, for each constant value of *μ*, the stability regions of the system shrink by decreasing the density gradient parameter. According to Figure 10b, since increasing the nonlocal parameter displaces the stability borders toward the lower velocities, one can assert that any increment in *μ* leads to a softer system. In other words, the nonlocal parameter induces a stiffness-softening effect on the system. Additionally, it can be observed that in contrast to the *v*_d_–*k*_f_ and *v*_d_–*α*_E_ curves, the *v*_d_–*μ* and *v*_d_–*α_ρ_* curves are overall descending, as *μ* and *α_ρ_* increase. Any increment in the nonlocal and density gradient parameters makes the system more unstable and leads to a diminishing of critical divergence velocity. Based on Figure 10a,b, for *α_ρ_* > 1, the stability of the system was enhanced by increasing *k*, whereas, this trend was the reverse for *α_ρ_* < 1. It should be mentioned that choosing a density gradient parameter close to one (i.e., homogeneous condition), the stability boundaries converge to each other for different power indices. In other words, for higher and lower values of the density gradient, the effect of the power index variations on the stability boundaries is more tangible. Another important point in the stability map of the system is that for lower values of nonlocal parameter, the critical velocity of the nanobeam declines monotonically by the increment of the density gradient parameter, whereas, this trend is not true for higher values of *μ*.

**Figure 11:**
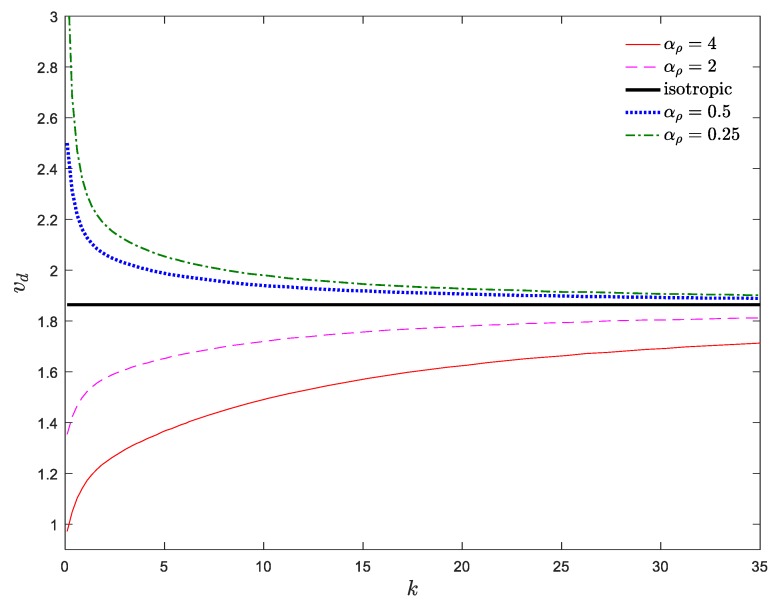
Dimensionless divergence velocity of a nanobeam against power index for *ζ* = 0, *α*_E_ = 1, *μ* = 0, *k*_f_ = 0.5.

Figure 11 demonstrates the stability map of the system in the *v*_d_–*k* plane. The indicated stability boundaries of the system in Figure 10 and Figure 11 are in agreement with each other. As is obvious, in the cases of density and elastic modulus grading, the power index has a reverse influence on the stability boundaries of the system. So, for *α_ρ_* > 1, an ascending trend is observed by increasing *k*, while, for *α_ρ_* < 1, this tendency is reversed. In other words, for *α_ρ_* > 1 (*α_ρ_* < 1), an increase of the power index leads to a more stable (unstable) structure. Moreover, similar to the case of elastic modulus grading, small values of *k* have a considerable impact on the stability regions of the axially moving AFG nanobeam, while the variation of the critical velocity of the structure in higher values of the power index is negligible. As a result, a small value of *k* plays an key role in the stability of axially moving AFG nanobeams. Generally, the simultaneous selection of lower values of *α_ρ_*, *k*_f_ and *k* is more suitable for the performance improvement of the system.

### 5.5. Effect of Simultaneous Density and Elastic Modulus Variations

**Figure 12:**
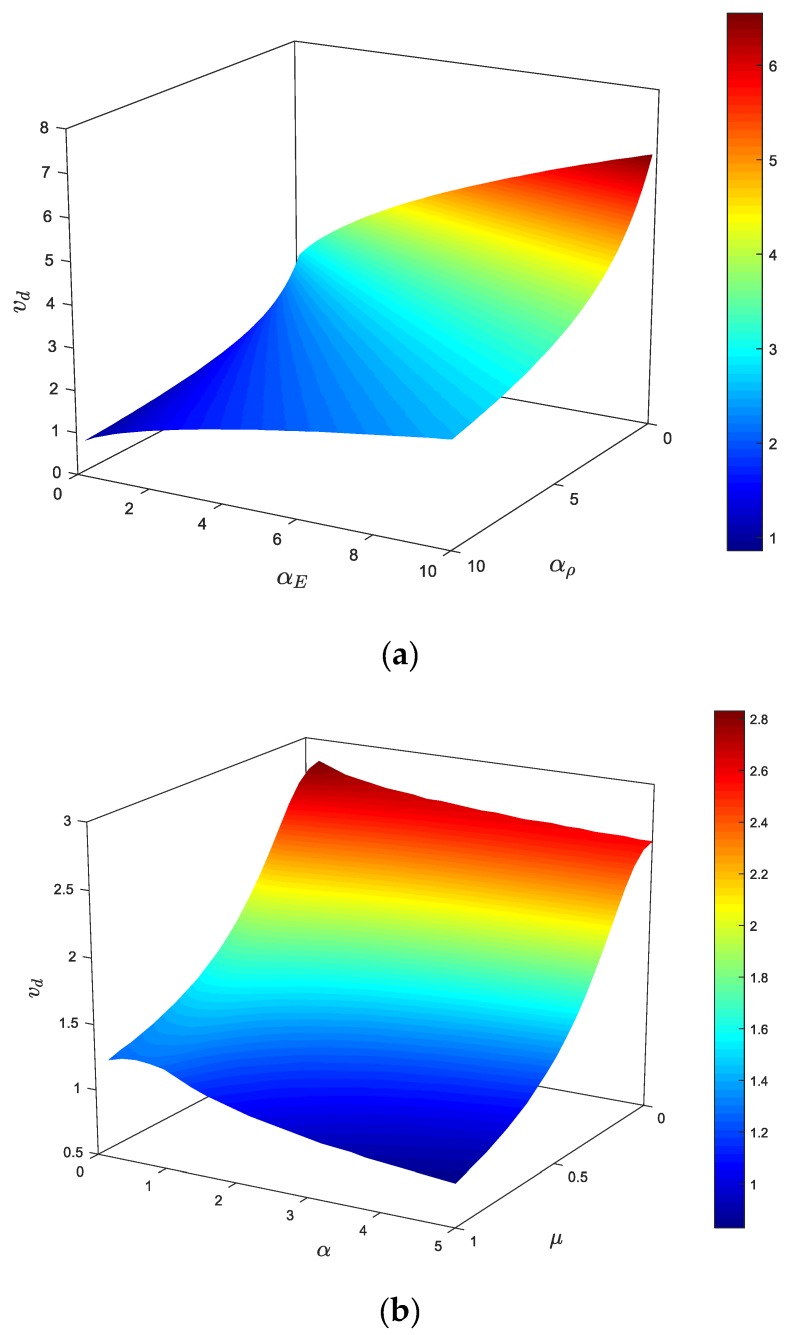
Combined effect of (**a**) density and elastic modulus gradient parameters and (**b**) nonlocal and gradient parameters on the critical velocity for *ζ* = 0, *k*_f_ = 0.5, *k* = 1.

**Figure 13:**
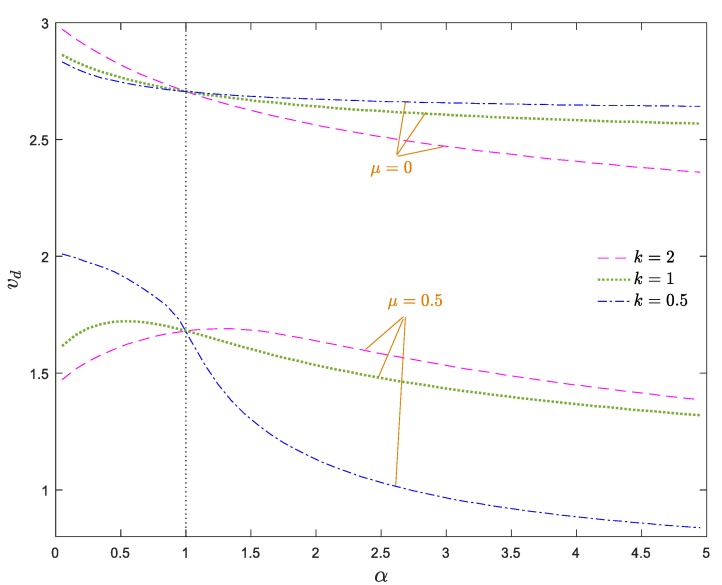
Dimensionless divergence velocity of the system against the gradient parameter for *ζ* = 0, *k*_f_ = 0.5.

Based on previous sections, it can be deduced that the variations of density and elastic modulus gradations along the axial direction play important roles in the vibrational response of the axially moving AFG nanobeams. Furthermore, it is demonstrated that the excessive vibrations of the system can be suppressed by adjusting *α_E_* and *α_ρ_* separately. According to Figure 6, Figure 7, Figure 8, Figure 9, Figure 10 and Figure 11, variations of density and elastic modulus along the longitudinal direction of the beam have opposite influences on the stability. In other words, decreasing the density gradient and ascending the elastic modulus gradient lead to an increase in the natural frequencies and expand the stability regions. As a result, these parameters can provide additional degrees of freedom to adjust the dynamic characteristics of axially moving nanosystems. In other words, it is possible to significantly improve the performance of axially moving nanosystems by simultaneous fine-tuning *α_E_* and *α_ρ_*. Therefore, determining the role of simultaneous gradation of the material properties on the stability of the moving nanobeams is of great importance. In this section, stability characteristics of the system are explored by considering the coupled density and elastic modulus variations through the axial direction (simultaneous mass-addition and stiffness-hardening effects). Additionally, the divergence velocity obtained through the Galerkin method can be evaluated by employing an analytical approach. To investigate the stability conditions of the system, the critical divergence velocity as a function of density and elastic modulus gradient parameters is plotted in Figure 12a. As shown in this figure, the critical velocity ascends by an increment of *α*_E_ and decrement of *α_ρ_*. In other words, the divergence strength of the system can be improved by decreasing *α_ρ_* and an increment of *α*_E_. For this reason, simultaneously choosing a higher elastic modulus gradient and a lower density gradient leads to a more stable structure, and consequently, better operational performance of axially moving nanostructures can be achieved. In Figure 12b, the instability thresholds of the structure when the density and elastic modulus gradient parameters are equal (*α*_E_ = *α_ρ_* = *α*) are illustrated. Generally, increasing the nonlocal parameter decreases the stability of the structure. Additionally, increasing the material gradient parameter (*α*) leads to a slight decline in the critical velocity of the system. Accordingly, compared with the stability maps presented in previous sections, one can deduce that the density gradient (mass-addition effect) plays a dominant role in the stability condition of the system, and the elastic modulus gradient has less impact on the dynamical behavior of the system. According to Figure 13, one can deduce that by fine-tuning the AFG material characteristics, the divergence threshold of the axially moving system could be significantly improved.

In Figure 13, the divergence velocity of the system versus the material gradient parameter is diagrammed for different values of power index. As depicted, the critical divergence velocities of the system obtained by the Galerkin method are in close agreement with those obtained by the analytical method presented in the Appendix A. Based on Figure 13, when *μ* = 0, the stability of the system declines monotonically by increasing *α*. Additionally, for *α* < 1, a decrease of *k* expands the stable regions, while for *α* > 1, a decrease of *k* leads to the shrinking of the stable regions. For higher values of *μ*, this trend reverses, so for *α* > 1 and *α* < 1, the decrement of the power index has a stabilizing and destabilizing effect on the system. Furthermore, when the nonlocal effects are highlighted in the system, the stability of the system varies non-monotonously with increasing *α*. It is worth noting that the influence of power index variation on the stability boundaries is not sensible for values of *α* close to one.

**Figure 14:**
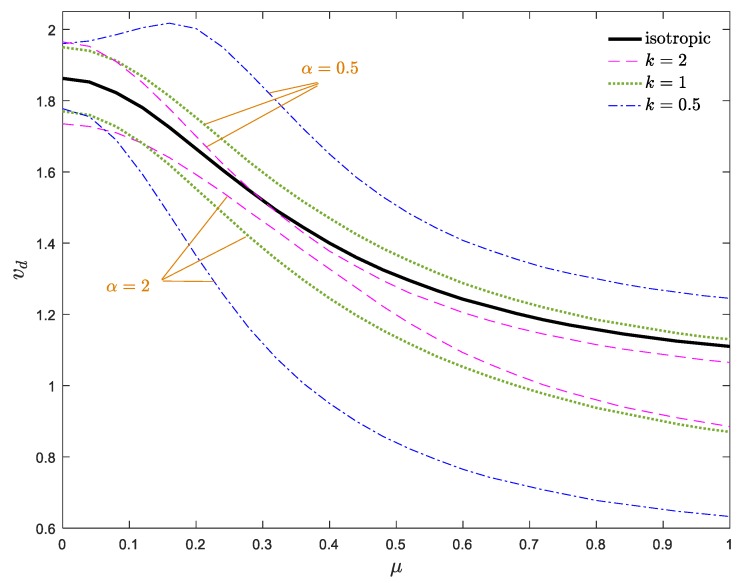
Critical divergence velocity of the system against nonlocal parameter for *ζ* = 0, *k*_f_ = 0.5.

For a better description of the dynamical stability of the system, the critical divergence velocity versus the nonlocal parameter is depicted in Figure 14. As can be seen, the divergence velocity of the system decreases by ascending the nonlocal parameter. The effect of the power index on the stability regions may be different, depending on the high and low values of the nonlocal parameter. For low values of *α* and *μ* or high values of *α* and *μ*, ascending the power index enhances the stability whereas, choosing a small power index for other conditions improves the performance of the system. Therefore, it can be concluded that the undesirable nonlocal effects can be alleviated by fine-tuning the materials distribution along the axial direction.

### 5.6. Effect of Viscoelastic Materials

**Figure 15:**
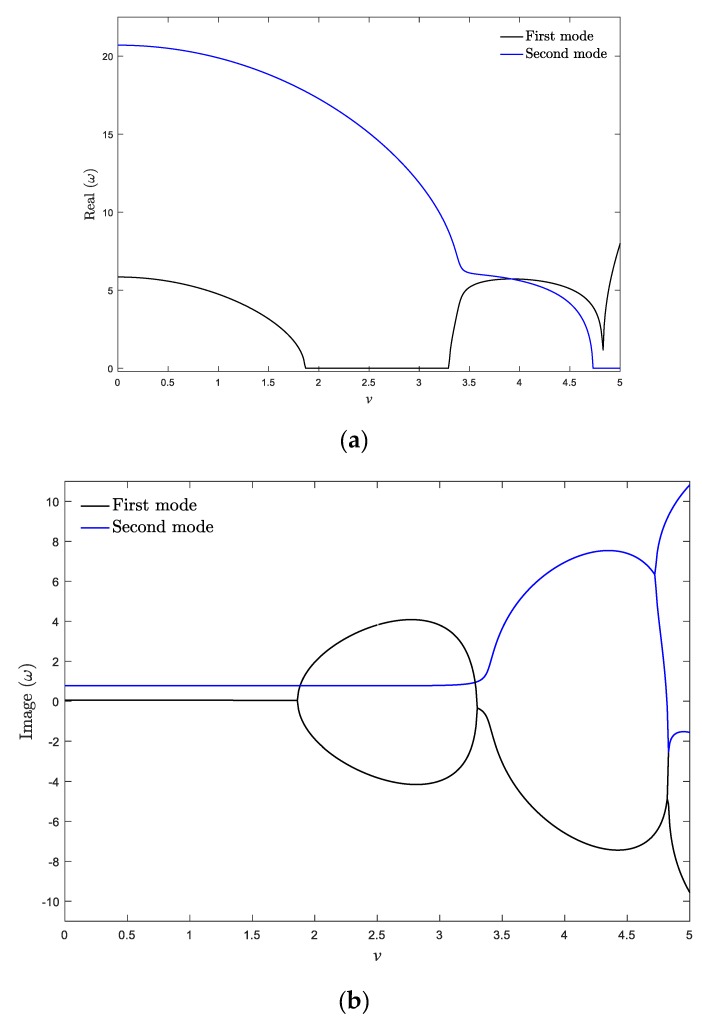
(**a**) Real and (**b**) imaginary parts of two natural frequencies of a viscoelastic moving isotropic nanobeam for *k*_f_ = 0.5, *μ* = 0.025, *ζ* = 0.001.

Finally, to explore the effect of viscoelastic materials on the dynamics of the system, real and imaginary parts of two first vibrational frequencies of the nanobeam versus axial velocity is demonstrated in Figure 15a,b, respectively, for *ζ* = 0.001 and *k*_f_ = 0.5. As shown, the critical divergence velocity of the first mode does not change by increasing the viscosity coefficient. This feature can be confirmed by the analytical approach existing in the Appendix A. Since the viscoelastic system is non-conservative, the natural frequencies are complex before the divergence, especially the frequency of higher modes. According to Figure 15b, the imaginary parts of the frequency curves lose their symmetry toward the *x*-axis in the case of the viscoelastic system. It can be seen that the viscoelastic system experiences the stability evolution of “stable—first mode flutter—second mode divergence”. Additionally, the first two natural frequency branches do not merge to a single branch beyond the critical divergence velocity. As a result, it can be concluded that compared with the isotropic and axially graded systems, utilizing viscoelastic materials changes the stability evolution of the axially moving nanosystems. Generally, it can be stated that the qualitative stability of the axially moving nanobeams is dependent on the effect of viscoelastic materials, while the axial gradation of materials plays an important role in determining the quantitative values of the critical velocity and natural frequencies of the system.

## 6. Conclusions

Structural dynamics and instability thresholds of axially moving viscoelastic AFG nanobeams were studied analytically and numerically in this study. The distribution of the material properties of the AFG system along the axial direction was considered according to the power-law function. Natural frequencies, dynamical response, and divergence and flutter instability thresholds of the system were obtained by Laplace transformation and the Galerkin discretization scheme to investigate the coupled effects of nanobeam velocity, dimensionless flexural stiffness, nonlocal parameter, viscosity coefficient, and axial material gradation parameters. Additionally, the accuracy of the presented solution approach was examined analytically. The main results of the current investigation can be listed as follows:

1. Compared with axially moving isotropic nanobeams, the system is more stable when along the axial direction, the density and elastic modulus decreases and increases, respectively. In other words, increasing the density and the elastic modulus gradient parameters have destabilizing and stabilizing effects on axially moving nanobeams, respectively
2. The greater the flexural stiffness, the more stable the system becomes, while a higher nonlocal parameter leads to a more unstable system. Additionally, the influence of the axial gradation of materials on the stability boundaries of the system is more tangible for high values of flexural stiffness.
3. The effect of the density gradient on the dynamical configuration of the system is dominant in the case of simultaneous axial variation of the density and modulus.
4. Increment/decrement of the power index leads to a more stable system when density and elastic modulus increase/decrease and decrease/increase through the axial direction, respectively.
5. Compared with axially moving isotropic and AFG nanosystems, employing viscoelastic materials in the system can change the stability evolution.

## Appendix A

The normalized eigenfunction of a simply-supported nonlocal beam is given by [54]:

(A1) $$\varphi_{r}\left(\xi \right)=\sqrt{2}\sin\left(\sigma_{r}\xi \right)$$

where

(A2) $$\sigma_{r}=q_{r}\sqrt{\frac{\sqrt{4+{\left(\mu q_{r}\right)}^{4}}+{\left(\mu q_{r}\right)}^{2}}{2}}$$

The characteristic frequency equation of the simply-supported boundary condition is as follows:

(A3) $$\sin\left(\sigma_{r}\right)=0$$

Based on the stability theory introduced by Lancaster [56], when the eigenvalue of the system equals zero, the system experiences the buckling phenomenon. In this case, the fundamental frequency of the system equal to zero when the system stiffness of the main mode becomes zero. As a result, the critical divergence velocity of the system is related to the first natural mode and Equation (16) reduces to the following equation by utilizing Equation (A1) and considering (*r* = *s* = 1):

(A4) $$M_{11}{\ddot{q}}_{1}\left(\tau \right)+C_{11}{\dot{q}}_{1}\left(\tau \right)+K_{11}q_{1}\left(\tau \right)=0$$

where the subscript one represents the first natural mode and the system stiffness related to the first mode may be written as:

(A5) $$k_{11}=-\frac{1}{\left(3+k\right)\left(k+1\right)\left(2+k\right)}\left(\begin{array}{l} \left(\begin{array}{l} -6\left(2+k\right)\left(\mu^{2}\left(\alpha_{\rho }-1\right)\nu^{2}\right)k\sigma_{1}^{3}\left(3+k\right)hypergeom\left(\left[1,\frac{1}{2}+\frac{1}{2}k\right],\left[\frac{3}{2},2,\frac{3}{2}+\frac{1}{2}k\right],-\sigma_{1}^{2}\right)+\\ 2\left(\left(\alpha_{\rho }-1\right)\nu^{2}-\nu_{f}^{2}\sigma_{1}^{2}\left(\alpha_{E}-1\right)\right)\left(k+1\right)\sigma_{1}^{3}hypergeom\left(\left[1,\frac{3}{2}+\frac{1}{2}k\right],\left[\frac{3}{2},2,\frac{5}{2}+\frac{1}{2}k\right],-\sigma_{1}^{2}\right)+\\ \left(\begin{array}{l} \left(\frac{7}{2}\sin\left(2\sigma_{1}\right)\left(\mu^{2}\left(\alpha_{\rho }-1\right)\nu^{2}k^{2}+\left(5\mu^{2}\left(\alpha_{\rho }-1\right)\nu^{2}\right)k\right)\sigma_{1}^{\frac{3}{2}-k}\right)2^{\frac{1}{2}-k}\\ -\frac{1}{2}2^{\frac{3}{2}-k}k^{2}\left(\sin\left(2\sigma_{1}\right)\nu_{f}^{2}\left(\alpha_{E}-1\right)\sigma_{1}^{\frac{3}{2}-k}+\nu^{2}\cos\left(2\sigma_{1}\right)\sigma_{1}^{\frac{1}{2}-k}\left(\alpha_{\rho }-1\right)\right) \end{array}\right) \end{array}\right)\\ LommelS1\left(\frac{3}{2}+k,\frac{1}{2},2\sigma_{1}\right)+7\left(\begin{array}{l} \left(\begin{array}{l} \left(\mu^{2}\left(\alpha_{\rho }-1\right)\nu^{2}-\frac{2}{7}\nu_{f}^{2}\left(\alpha_{E}-1\right)\right)\left(2+k\right)\left(3+k\right)\sigma_{1}^{\frac{5}{2}-k}+\\ \frac{5}{7}\left(k+\frac{6}{5}\right)\nu^{2}\left(\alpha_{\rho }-1\right)\sigma_{1}^{\frac{1}{2}-k} \end{array}\right)2^{\frac{1}{2}-k}+\\ \frac{1}{14}\nu^{2}2^{\frac{3}{2}-k}\sigma_{1}^{\frac{1}{2}-k}k^{2}\left(\alpha_{\rho }-1\right) \end{array}\right)\\ k\sin\left(2\sigma_{1}\right)LommelS1\left(k+\frac{1}{2},\frac{3}{2},2\sigma_{1}\right)-\frac{1}{2}\nu^{2}\left(\alpha_{\rho }-1\right)\mu^{2}\left(\begin{array}{l} -\cos\left(2\sigma_{1}\right)\left(3+k\right)\left(k+1\right)\left(2+k\right)\sigma_{1}^{\frac{3}{2}-k}\\ +3\sigma_{1}^{\frac{1}{2}-k}\sin\left(2\sigma_{1}\right)\left(k^{2}+1\right) \end{array}\right) \end{array}\right)$$
